# Spatial Characteristics of the Efflux Pump MexB Determine Inhibitor Binding

**DOI:** 10.1128/aac.00672-22

**Published:** 2022-10-27

**Authors:** Seiji Yamasaki, Naoki Koga, Martijn Zwama, Keisuke Sakurai, Ryosuke Nakashima, Akihito Yamaguchi, Kunihiko Nishino

**Affiliations:** a Institute for Advanced Co-Creation Studies, Ibaraki, Osaka, Japan; b Graduate School of Pharmaceutical Sciences, Osaka University, Suita, Osaka, Japan; c SANKEN (The Institute of Scientific and Industrial Research), Osaka University, Ibaraki, Osaka, Japan; d Center for Infectious Disease Education and Research, Osaka University, Suita, Japan

**Keywords:** RND efflux pumps, antibiotic resistance, antibiotics, efflux pump inhibitors, molecular dynamics, molecular modeling, multidrug efflux pumps, multidrug resistance, multidrug transporter

## Abstract

The multidrug efflux transporters MexB and MexY in Pseudomonas aeruginosa and AcrB in Escherichia coli contribute to these organisms’ multidrug resistance. Efflux pump inhibitor (EPI) ABI-PP inhibits MexB and AcrB, but not MexY. We previously determined the structure of ABI-PP bound to the hydrophobic trap (the inhibitor-binding pit) of AcrB and MexB. The insensitivity of MexY to ABI-PP was attributed to a bulky tryptophan (Trp). AcrB(Phe178Trp) became uninhibited by ABI-PP, while MexY(Trp177Phe) resensitized MexY for ABI-PP. Interestingly, ABI-PP was able to inhibit MexB(Phe178Trp). Thus, it is not clear which bulky amino acid mutations are critical for inhibitor binding in MexB. Here, we investigated the pit of MexB in more detail, and elucidated which Trp mutation locations in the pit were hindering ABI-PP binding, but did not affect the function of the efflux pumps. Mutating positions 139, 277, 279, and 612 to tryptophan eliminated the inhibitory effect. However, the tryptophan mutation at position 571 did not cause any effect. These results show that the effectiveness of EPIs is greatly affected by mutations in different locations, and that binding of EPIs is partly attributed by spatial characteristics. These results should be taken into account for new inhibitor and drug discovery.

## INTRODUCTION

Bacteria express multidrug transporters responsible for virulence, biofilm formation, and resistance to toxic compounds and metabolites (1–8). When overexpressed, multidrug efflux transporters can cause multidrug resistance (MDR) (9), as these single pumps can recognize and discharge a wide range of antibacterial agents (10, 11). Therefore, multidrug efflux transporters have been important targets for the development of novel antibiotics and efflux pump inhibitors (EPIs). In Gram-negative bacteria, efflux pumps belonging to the resistance-nodulation-division (RND) superfamily are the main contributors to MDR, pumping drugs across the inner and outer membranes (12, 13). The major and most studied RND-type efflux pump in Escherichia coli is AcrB (14). In Pseudomonas aeruginosa, the major efflux pumps are MexY and MexB (15, 16). These pumps are responsible for both acquired antibiotic resistance and intrinsic resistance to toxic compounds (such as bile salts and fatty acids) (8). RND-type pumps are present in all bacteria and archaea (17), and we recently studied the phylogenetic relationship between a multitude of these pumps and their inhibitor-binding pits (18).

We solved the first symmetrical crystal structure of an RND-type efflux pump, which was E. coli AcrB (19). Later crystal structures revealed AcrB in its asymmetric homotrimeric state, bound with drug molecules (20–22). Each of the three monomers presented different conformations: access, binding, and extrusion. They revealed that AcrB moves drugs from a substrate binding pocket (the distal binding pocket, or DBP) to an exit funnel through the “functionally rotating mechanism” (20). We later found a second voluminous drug binding pocket (the proximal binding pocket, or PBP) (23), which is placed tandemly before the DBP, being part of a drug translocation route. Drugs are translocated by a peristaltic motion (21), in which drugs are translocated through the monomer by the alternately expansion and contraction of the PBP and the DBP (14).

We later solved the first crystal structures of RND pumps bound with an EPI: pyridopyrimidine derivative ABI-PP (24). ABI-PP is [[2-({[((3*R*)-1-{8-{[(4-*tert*-butyl-1,3-thiazol-2-yl)amino]carbonyl}-4-oxo-3-[(*E*)-2-(1*H*-tetrazol-5-yl)vinyl]-4*H*-pyrido[1,2-*a*]pyrimidin-2-yl}piperidin-3-yl)oxy]carbonyl}amino) ethyl](dimethyl) ammonio] acetate (D13-9001) (25). This EPI inhibited the functionality of certain pumps completely. ABI-PP (and other EPIs) (26–28) was bound tightly in AcrB (E. coli) and MexB (P. aeruginosa), located in a branched-off hydrophobic trap of the DBP. This inhibitor-binding pit is rich in phenylalanine residues, interacting with the EPI molecules (24, 26–28). However, ABI-PP was unable to inhibit MexY (24). Our study (24) showed that steric hindrance by a bulky tryptophan residue at the center of the MexY hydrophobic trap was causing significant steric hindrance, making it impossible for ABI-PP to bind and subsequently inhibit active drug export. When introducing a tryptophan mutation (F178W) in AcrB, AcrB became insensitive to ABI-PP, while maintaining active drug export. Similarly, when the tryptophan in MexY was mutated to less bulky phenylalanine (W177F), MexY became sensitive to ABI-PP. Interestingly, the tryptophan mutation (F178W) in MexB did not affect the inhibitory effect. Thus, it is not clear which bulky amino acid mutations are critical for inhibitor binding in MexB. Therefore, we investigated the spatial characteristics of the MexB hydrophobic trap for EPI effectiveness, by introducing steric hindrances. Here, we demonstrated that amino acid substitution at alternative regions within this pit could confer ABI-PP resistance in MexB. These results are important for the developments of novel EPIs.

## RESULTS

### Structural analysis of the MexB hydrophobic trap.

**FIG 1:**
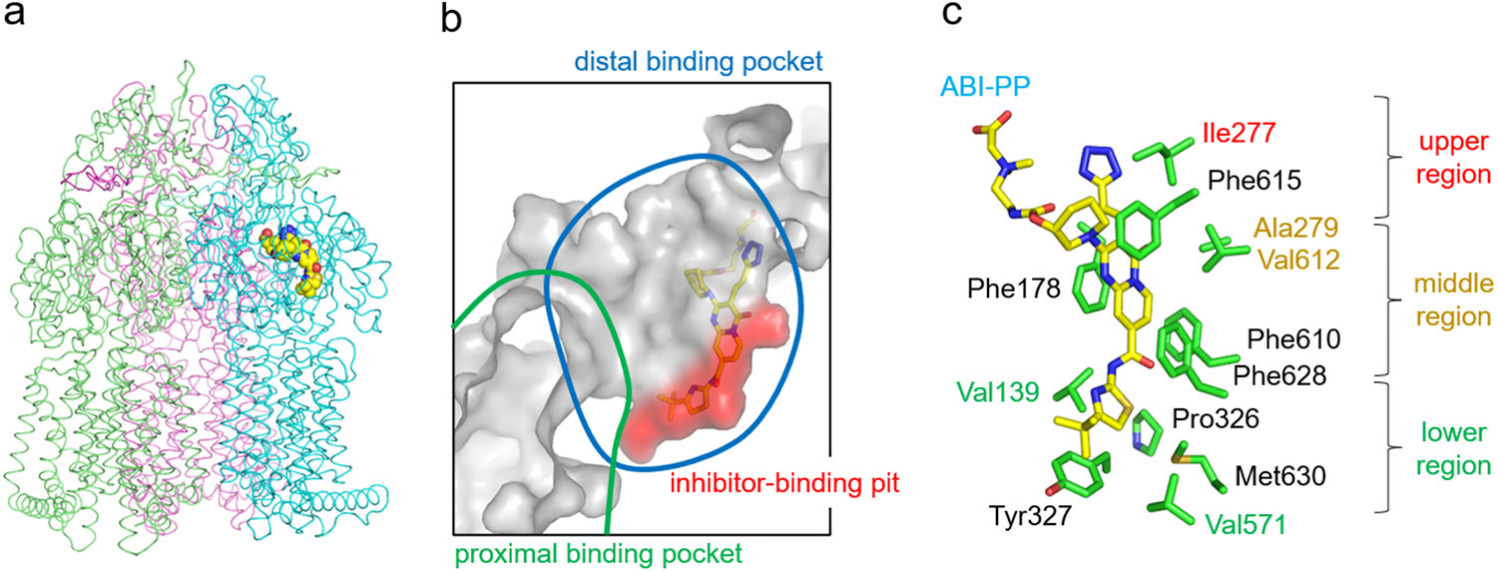
Inhibitor-binding site of wild-type MexB. (a) Crystal structure of the inhibitor ABI-PP bound to the MexB trimer. Three MexB monomers are shown in green, blue, and red, representing the access, binding, and extrusion monomer, respectively. ABI-PP is shown as the yellow space-filling model. (b) Close-up view of the inhibitor-binding site. Substrate translocation pathway is shown as a solid gray surface. The proximal and distal binding pockets are indicated in green and blue circles, respectively. The inhibitor-binding pit is shown as a red surface. The ABI-PP molecule is represented as a yellow stick model. (c) Detailed inhibitor-binding site. Carbon atoms of ABI-PP and the amino acid residues are indicated in yellow and green, respectively. The classification of the amino acids is shown on the right side of the panel.

To determine the special characteristics of the MexB efflux pump in regard to the ability of ABI-PP to bind to the hydrophobic pit, we introduced bulky tryptophan residues (Trp) at various locations, and checked which locations caused a steric clash with ABI-PP, and which did not. The requirement for a successful mutation was that the transporter needed to function similar to wild-type MexB. Fig. 1a shows the crystal structure of MexB and Fig. 1b shows the location of ABI-PP within the binding monomer (24). Investigation of the ABI-PP molecule in the hydrophobic trap (Fig. 1c) provided us with the amino acids with side chains directed toward ABI-PP. Phenylalanine residues (Phe) were excluded from the mutation candidates because the substitution from Phe to Trp is a sterically less significant alteration and causes less steric hindrance than the substitution of other residues to Trp. The initially chosen candidate residues were Val139, Phe178, Ile277, Ala279, Pro326, Tyr327, Val571, Phe610, Val612, Phe615, Phe628, and Met630 (Fig. 1c). All of these residues are also conserved between AcrB and MexB (Fig. S1). Among these amino acids, Val139, Ile277, Ala279, Val571, and Val612 were selected to be mutated to tryptophan in this study, as we believed these mutations would sterically hinder ABI-PP most significantly. We classified the residues around the ABI-PP molecule into three regions, namely: upper (Ile277), middle (Ala279 and Val612), and lower (Val139 and Val571) as shown in Fig. 1c.

### Bulky mutations in the upper and middle regions prevent inhibitor binding.

**FIG 2:**
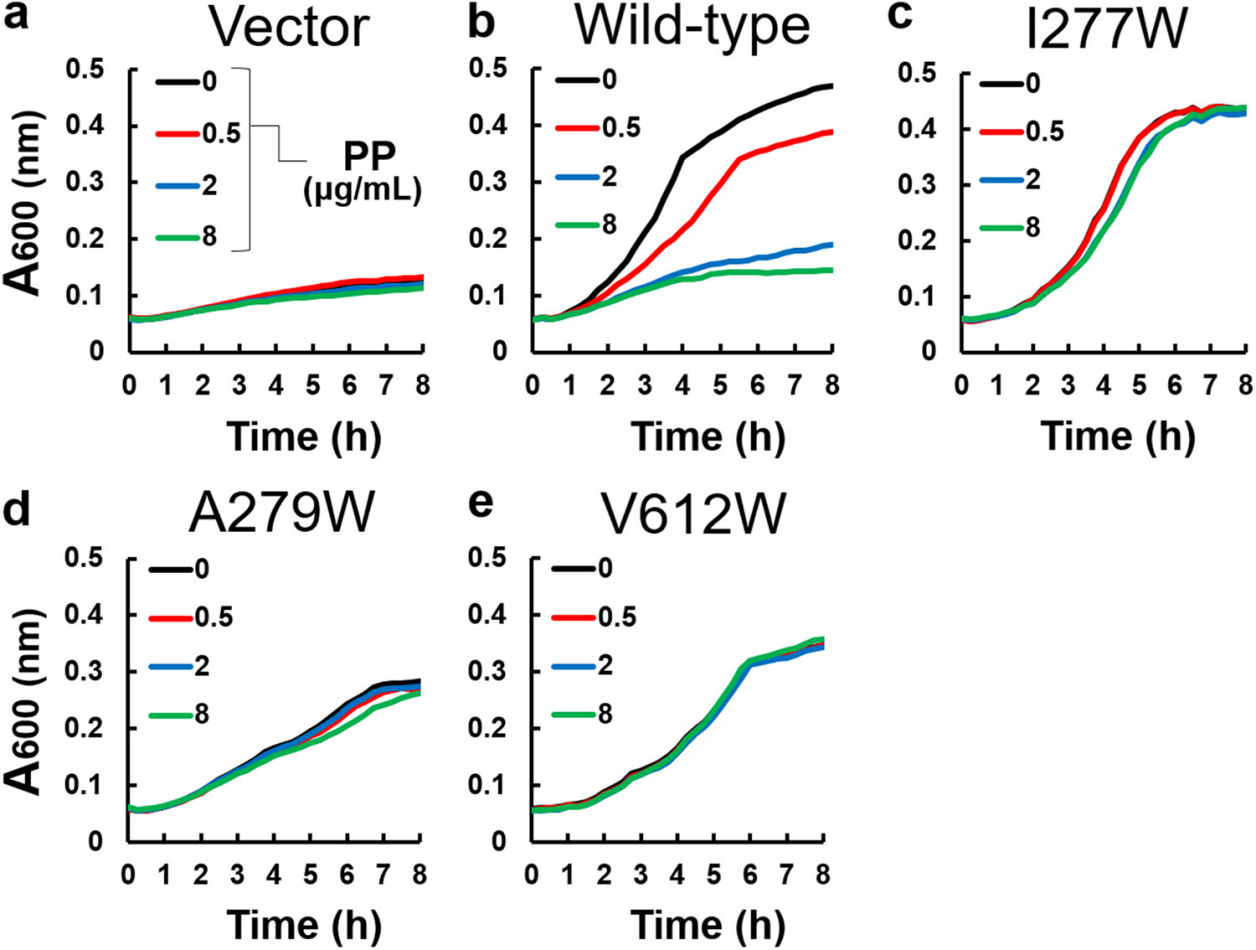
The inhibitory effect of ABI-PP on the upper and middle regions mutants. (a) The growth of E. coli MG1655Δ*acrB*Δ*tolC* cells harboring the plasmid pMMB67HE in the presence of 3 μg/mL of erythromycin and various concentrations of ABI-PP. (b to e) The growth of E. coli MG1655Δ*acrB*Δ*tolC* expressing wild-type MexB (b), MexB(I277W) (c), MexB(A279W) (d), and MexB(V612W) (e) in the presence of 3 μg/mL of erythromycin and various concentrations of ABI-PP. These tests were performed in triplicates. PP, ABI-PP inhibitor.

Ile277 is located near the entrance of the pit, which is branched from the distal binding pocket, and its side chain is close to the tetrazole moiety of the ABI-PP molecule (Fig. 1c). Ala279 and Val612 are located in the middle region of the pit, close to the pyridopyrimidine bicyclic aromatic moiety of ABI-PP (Fig. 1c). Plasmids carrying the *mexABoprM* gene (with the relevant mutations) were transformed into Escherichia coli MG1655Δ*acrB*Δ*tolC* cells, and the MexB efflux pumps were tested on their functionality by observing bacterial growth in liquid medium, supplemented with and without each antibiotic (3 μg/mL erythromycin, 0.016 μg/mL levofloxacin, and 0.125 μg/mL aztreonam). The Δ*acrB*Δ*tolC* cells expressing wild-type MexB could grow in the presence of erythromycin (Fig. 2b), while the same cells carrying an empty vector could not (Fig. 2a), as found previously (24). The addition of ABI-PP in various concentrations in the absence of erythromycin did not affect cell growth, when the mutated MexB proteins were expressed (Fig. S2). As a positive control, the addition of ABI-PP in various concentrations (0.5, 2 and 8 μg/mL) in the presence of erythromycin (3 μg/mL) decreased the growth of wild-type MexB expressing cells in a concentration dependent manner, with 8 μg/mL ABI-PP resulting in a complete inhibition of the active transport of the antibiotic (Fig. 2b). However, when ABI-PP was added to the upper and middle Trp-mutated MexB (I277W, A279W, and V612W) expressing cells, the cell growth in the presence of erythromycin was unaffected (Fig. 2c to e). The results for levofloxacin and aztreonam showed almost the same trend as for erythromycin (Fig. S3 and 4). These suggest that bulky Trp mutations at the entrance or middle region of the inhibitor binding pit prevent the binding of the inhibitor ABI-PP.

### Lower regions of the pit are less sensitive to Trp substitutions.

**FIG 3:**
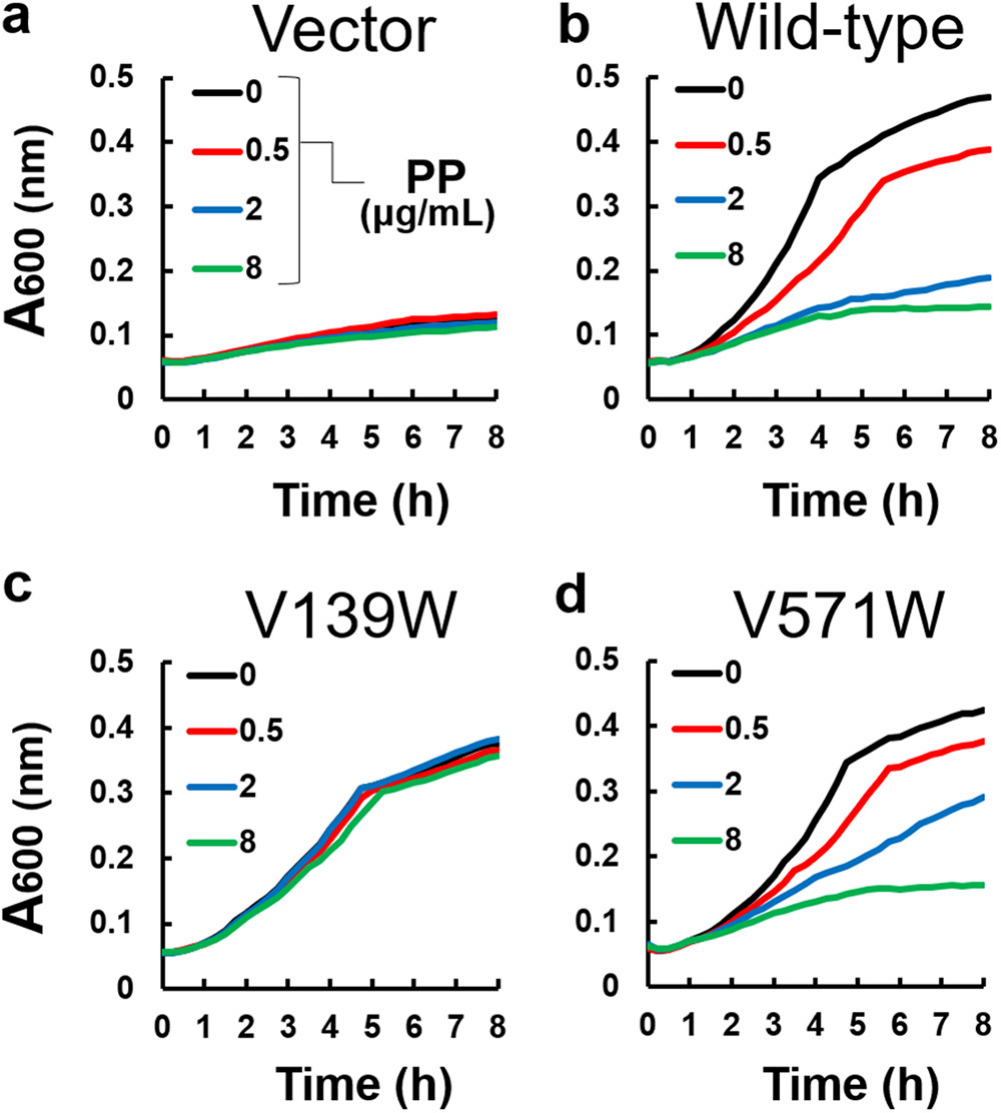
The inhibitory effect of ABI-PP on the lower region mutants. (a) The growth of E. coli MG1655Δ*acrB*Δ*tolC* cells harboring the plasmid pMMB67HE in the presence of 3 μg/mL of erythromycin and various concentrations of ABI-PP. (b to d) The growth of E. coli MG1655Δ*acrB*Δ*tolC* expressing wild-type MexB (b), MexB(V139W) (c), and MexB(V571W) (d) in the presence of 3 μg/mL of erythromycin and various concentrations of ABI-PP. These tests were performed in triplicates. PP, ABI-PP inhibitor.

**FIG 4:**
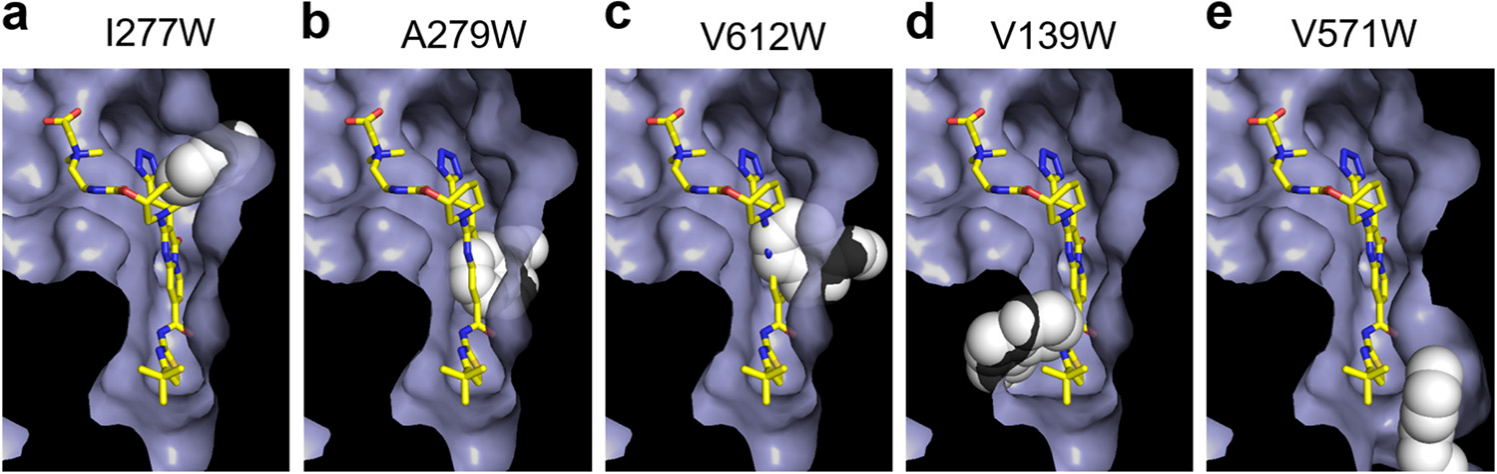
Evaluation of the putative structures of MexB mutants. (a to e) Modeled closeup views of the inhibitor-binding pit of ABI-PP-bound MexB mutants I277W (a), A279W (b), V612W (c), V139W (d), and V571W (e). These binding-pits are shown as solvent accessible surface, except for the tryptophan. ABI-PP and tryptophan side chains are shown by stick and space-filling model, respectively. The carbon atoms of ABI-PP and mutated tryptophan are indicated in yellow and white, respectively.

Val139 and Val571 are located at the lower region of the inhibitor binding pit (Fig. 1c). The side chains of these residues are in the vicinity of the pyrrole and isobutane moieties of ABI-PP (Fig. 1c). When Val139 was mutated to Trp, the MexB (V139W)-expressing E. coli cells could grow in the presence of erythromycin, and were (similar to the upper and middle mutations) unaffected by the addition of ABI-PP (Fig. 3c). On the other hand, the Trp substitution at Val571 gave similar results to wild-type MexB (Fig. 3d). Here, the addition of ABI-PP too decreased the cell growth in a concentration dependent manner. The results for levofloxacin and aztreonam showed almost the same trend as for erythromycin (Fig. S3 and 4). Computational analysis was performed to analyze the tryptophan mutations in the MexB hydrophobic pit. Tryptophan orientations were adjusted manually (see Materials and Methods). To avoid collision of tryptophan with the protein at the positions Val139, Ile277, and Val571, it was necessary to apply torsion angle adjustment to Phe136, Arg620, and Phe573/Phe517, respectively. The A279W mutation required not only a rotation of Phe610 and the tryptophan side chain, but also slight modification of ∠Cα-Cβ-Cγ of the tryptophan. The V612W mutation could be made by only rotation itself. As a result, the bulky tryptophan side chains significantly decreased the space of the inhibitor binding pit for the upper and middle regions (Fig. 4a to c). Tryptophan side chains in I277W mutated MexB significantly narrowed the entrance of the pit (Fig. 4a), and A279W and V612W clashed with the pyridopyrimidine rings (Fig. 4b and c). In the case of the lower pit mutations, V139W significantly narrowed the already narrow region of the pit (Fig. 4d), and the Trp side chain clashed in the simulation significantly with the ABI-PP molecule. However, V571W did not cause a clash with the ABI-PP inhibitor, and there was enough space of the bulky side chain to move aside and be distanced from the inhibitor (Fig. 4e).

## DISCUSSION

In our previous study (24), we identified amino acids tightly binding ABI-PP to MexY and MexB (the major efflux pumps in P. aeruginosa) and AcrB (the main efflux pump in E. coli). In the case of MexY, ABI-PP was only binding when a bulky Trp present in the hydrophobic trap was altered to Phe. The location of this Trp was W177, which corresponds to the Phe residue F178 in both MexB and AcrB. Introducing the Trp mutation at this location resulted in an ABI-PP resistant AcrB, in contrast to MexB, which remained sensitive to ABI-PP. Amino acid mutations (F628L and ΔV177) that affect the ABI-PP susceptibility of MexB were found by selection studies (26), but it is not clear which bulky mutations are critical. The MexB variants in this study (V139W, I277W, A279W, V571W, and V612W) could all actively efflux antibiotics, as suggested by the growth ability. ABI-PP could inhibit bacterial growth under each antibiotic supplemented conditions (indicating the inhibition by the EPI) for wild-type MexB. In contrast to the F178W mutation, we found that MexB lost sensitivity to ABI-PP in all mutants, except for V571W. Investigation indicated that there is no available space for ABI-PP to bind in the hydrophobic trap of MexB variants V139W, I277W, A279W, and V612W, because of the space-filling side chain. In contrast, in the V571W variant, tryptophan was positioned in such a way that it did not interfere with ABI-PP binding due to the arrangement with neighboring amino acids, similar to what we previously found for the F178W mutant.

The crystal structure of ABI-PP (24) to AcrB and MexB and other EPIs (27, 28) to AcrB indicate a strong binding of Phe residues (especially Phe178 and Phe628) (27) to the EPI molecules. However, not only are the Phe residues important for binding by π-π stacking, so are residues such as Tyr327 (AcrB and MexB) and Met573 (AcrB, corresponding to Phe573 in MexB) (24, 27). Despite most Phe residues and residues of interested in this study being conserved (Fig. S1) between AcrB and MexB (Val139, Phe178, Ile277, Ala279, Pro326, Tyr327, Val571, Phe610, Val612, Phe615, and Phe628) (18, 24), the ability of ABI-PP binding is not only determined by these residues. MexB and AcrB have significantly conserved hydrophobic traps (especially compared with their drug binding pockets) (18); however, a single mutation within MexY (W177F) rendered this pump sensitive to the ABI-PP inhibitor, despite MexY having a less conserved hydrophobic trap compared with AcrB and MexB (according to sequence alignment). For example, the Phe residues in the conserved pockets of AcrB and MexB are substituted in MexY by Ile, Trp, Tyr, and Leu residues (18). The determinants of inhibitor and substrate binding to RND-type efflux pumps have been under long debate and investigation (20, 21, 23, 24, 29–38). An interesting mutation F610A within the hydrophobic trap of AcrB resulted in a mostly inactive mutant (39), while at the same time, a bulky charged mutation (F610E) resulted in a completely active transporter (18). This region is not the determinant binding pocket for many substrates in the transporter (40), corroborating how delicate the recognition is, based not only on direct amino acid interactions, but also by, e.g., local hydrophobicity and electrostatic potential (29). In contrast to the pumps’ export substrates, ABI-PP (and other pyranopyridine-derived EPIs) is tightly bound and fixed in one location of one monomer (24), and does not oscillate between different locations or bind loosely to different subsets of residues (14, 27).

Our research shows that bulky alterations not only in the middle region of the pit, but also at various neighboring locations adjacent to the inhibitor-binding site, prevent EPI binding and spatial properties of the entirety of inhibitor-binding pit and are important for the effectiveness of EPIs. Therefore, hypothetically, when EPIs are used in a clinical setting, a mutation resulting in EPI resistance would not necessarily give clinically relevant insights into a specific location useful to predict future EPI resistance in other or identical multidrug efflux pumps. We show that the spatial characteristics of the entire pit determines the effectiveness of an EPI. Analysis of EPI-resistant mutations in multidrug efflux pumps is important for the development of novel inhibitors.

## MATERIALS AND METHODS

### Bacterial strains, plasmids, and growth conditions.

The bacterial strains and plasmids used in this study are listed in Table 1. E. coli strains were derived from the wild-type strain MG1655 (41). To construct the Δ*acrB*Δ*tolC* mutant, gene deletion was performed following the method of Datsenko and Wanner (42). Drug resistance markers were eliminated using the plasmid pCP20. The pMMB67HE vectors carrying the *mexABoprM* genes were transformed into MG1655Δ*acrB*Δ*tolC* cells. Bacterial strains were cultured at 37°C in Luria-Bertani broth (43).

**TABLE 1:** Escherichia coli strains and plasmids used in this study

Strain or plasmid	Characteristics	Source or reference
Strains		
MG1655	Wild-type	41
NKE128	Δ*acrB*Δ*tolC*	45
NKE1629	Δ*acrB*Δ*tolC*/pMMB67HE	This study
NKE1630	Δ*acrB*Δ*tolC*/pMexAB^his^M	This study
NKE1801	Δ*acrB*Δ*tolC*/pMexAB^his^(V612W)M	This study
NKE1802	Δ*acrB*Δ*tolC*/pMexAB^his^(A279W)M	This study
NKE1803	Δ*acrB*Δ*tolC*/pMexAB^his^(V139W)M	This study
NKE1804	Δ*acrB*Δ*tolC*/pMexAB^his^(V571W)M	This study
NKE1805	Δ*acrB*Δ*tolC*/pMexAB^his^(I277W)M	This study
Plasmids		
pMMB67HE	Vector; SBPC^R^	46
pMexAB^his^M	*mexA, mexB,* and *oprM* genes cloned into pMMB67HE, SBPC^R^	44

### Site-directed mutagenesis.

The plasmid pMexAB^his^M (pMMB67HE containing the *mexABoprM* genes) was provided by T. Nakae (44). Point mutations were introduced by PCR using primers to create the following codon replacements: V139W (GTG→TGG), I277W (ATC→TGG), A279W (GCG→TGG), V571W (GTA→TGG), and V612W (GTG→TGG). The constructed plasmids were confirmed by sequencing using a 3100-Avant Genetic Analyzer (Applied Biosystems, Carlsbad, CA, USA). Plasmids were transformed in E. coli MG1655Δ*acrB*Δ*tolC* cells.

### Each antibiotic and ABI-PP susceptibility testing.

Single E. coli colonies carrying the pMexAB^his^M-derived plasmids were inoculated into 2 mL of LB broth and cultured overnight at 37°C. Then, 2 × 10^7^ CFU/μL of bacteria were inoculated into 200 μL of LB broth containing both or either each antibiotic and various concentrations of ABI-PP (0.5, 2, and 8 μg/mL). Liquid cultures were incubated and shaken at 37°C. Bacterial growth in 96-well plates was measured by OD_600nm_ readings using an Infinite M200 Pro Plate Reader (Tecan, Switzerland).

### Evaluation of the putative tryptophan mutation points.

Mutant models were made to evaluate the validity of the tryptophan mutation positions based on the crystal structure of ABI-PP bound MexB (PDB-ID: 3W9J) using *PyMOL* (Schrödinger, LLC). The steric hindrance resulting from the mutation is minimized by the torsion angle adjustment of the tryptophan and the neighbor amino acid side chain manually.

### Statistics and reproducibility.

Drug susceptibility experiments and the inhibition effectiveness experiments were repeated at least three times to validate the reproducibility.

### Data availability.

Data are available in this article and the supporting figures and tables are available as supplementary information files. Other data that support the findings of this study are available from the corresponding author upon request.
