# Supplementary material for: Spatial Characteristics of the Efflux Pump MexB Determine Inhibitor Binding
Source: Antimicrob Agents Chemother. 2022 Oct 27;66(11):e00672-22. doi: 10.1128/aac.00672-22 (PMC9664856; doi:10.1128/aac.00672-22)
Supplement: Supplemental file 1 — Supplemental material. Download aac.00672-22-s0001.pdf, PDF file, 0.7 MB [file aac.00672-22-s0001.pdf]

SUPPLEMENTARY INFORMATION

Spatial characteristics of the efflux pump MexB determine inhibitor binding

Seiji Yamasaki[a,b,c], Naoki Koga[b], Martijn Zwama[c], Keisuke Sakurai[c,e], Ryosuke Nakashima[c], Akihito Yamaguchi[c], and Kunihiko Nishino[b,c,d*]

[a]Institute for Advanced Co-Creation Studies, Ibaraki, Osaka 567-0047, Japan
[b]Graduate School of Pharmaceutical Sciences, Osaka University, Suita, Osaka 565-0871, Japan
[c]SANKEN (The Institute of Scientific and Industrial Research), Osaka University, Ibaraki, Osaka 567-0047, Japan
[d]Center for Infectious Disease Education and Research, Osaka University, Suita, Japan
[e]Present address. Institute for Protein Research, Osaka University, Suita, Osaka 565-0871, Japan

Supplementary Materials

Supplementary Figure 1. Alignment of *E. coli* AcrB and *P. aeruginosa* MexB.

Supplementary Figure 2. Growth of *Escherichia coli* cells in the presence of various concentrations of ABI-PP without antibiotics

Supplementary Figure 3. Growth of *Escherichia coli* cells in the presence of 0.016 µg/mL of levofloxacin and various concentrations of ABI-PP

Supplementary Figure 4. Growth of *Escherichia coli* cells in the presence of 0.125 µg/mL of aztreonam and various concentrations of ABI-PP

```
MexB  1     MSKFFIDRPIFAWVIALVIMLAGGLSILSLPVNQYPAIAPPAIAVQVSYPGASAETVQDT  60
            M  FFIDRPIFAWVIA++IMLAGGL+IL LPV QYP IAPPA+ +  SYPGA A+TVQDT
AcrB  1     MPNFFIDRPIFAWVIAIIIMLAGGLAILKLPVAQYPTIAPPAVTISASYPGADAKTVQDT  60

MexB  61    VVQVIEQQMNGIDNLRYISSESNSDGSMTITVTFEQGTDPDIAQVQVQNKLQLATPLLPQ  120
            V QVIEQ MNGIDNL Y+SS S+S G++ IT+TFE GTD DIAQVQVQNKLQLA PLLPQ
AcrB  61    VTQVIEQNMNGIDNLMYMSSNSDSTGTVQITLTFESGTDADIAQVQVQNKLQLAMPLLPQ  120

MexB  121   EVQRQGIRVTKAVKNFLMVVGVVSTDGSMTKEDLSNYIVSNIQDPLSRTKGVGDFQVFGS  180
            EVQ+QG+ V K+  +FLMVVGV++TDG+MT+ED+S+Y+ +N++D +SRT GVGD Q+FGS
AcrB  121   EVQQQGVSVEKSSSSFLMVVGVINTDGTMTQEDISDYVAANMKDAISRTSGVGDVQLFGS  180

MexB  181   QYSMRIWLDPAKLNSYQLTPGDVSSAIQAQNVQISSGQLGGLPAVKGQQLNATIIGKTRL  240
            QY+MRIW++P +LN +QLTP DV +AI+AQN Q+++GQLGG P VKGQQLNA+II +TRL
AcrB  181   QYAMRIWMNPNELNKFQLTPVDVITAIKAQNAQVAAGQLGGTPPVKGQQLNASIIAQTRL  240

MexB  241   QTAEQFENILLKVNPDGSQVRLKDVADVGLGGQDYSINAQFNGSPASGIAIKLATGANAL  300
             + E+F  ILLKVN DGS+V L+DVA + LGG++Y I A+FNG PASG+ IKLATGANAL
AcrB  241   TSTEEFGKILLKVNQDGSRVLLRDVAKIELGGENYDIIAEFNGQPASGLGIKLATGANAL  300

MexB  301   DTAKAIRQTIANLEPFMPQGMKVVYPYDTTPVVSASIHEVVKTLGEAILLVFLVMYLFLQ  360
            DTA AIR  +A +EPF P G+K+VYPYDTTP V  SIHEVVKTL EAI+LVFLVMYLFLQ
AcrB  301   DTAAAIRAELAKMEPFFPSGLKIVYPYDTTPFVKISIHEVVKTLVEAIILVFLVMYLFLQ  360

MexB  361   NFRATLIPTIAVPVVLLGTFGVLAAFGFSINTLTMFGMVLAIGLLVDDAIVVVENERVM  420
            NFRATLIPTIAVPVVLLGTF VLAAFGFSINTLTMFGMVLAIGLLVDDAIVVVENERVM
AcrB  361   NFRATLIPTIAVPVVLLGTFAVLAAFGFSINTLTMFGMVLAIGLLVDDAIVVVENERVM  420

MexB  421   AEEGLSPREAARKSMGQIQGALVGIAMVLSAVFLPMAFFGGSTGVIYRQFSITIVSAMAL  480
            AEEGL P+EA RKSMGQIQGALVGIAMVLSAVF+PMAFFGGSTG IYRQFSITIVSAMAL
AcrB  421   AEEGLPPKEATRKSMGQIQGALVGIAMVLSAVFVPMAFFGGSTGAIYRQFSITIVSAMAL  480

MexB  481   SVIVALILTPALCATMLKPIEKGDHGEHKAGFFGWFNRMFLSTTHGYERGVASILKHRAP  540
            SV+VALILTPALCATMLKPI KGDHGE K GFFGWFNRMF  +TH Y   V  IL+
AcrB  481   SVLVALILTPALCATMLKPIAKGDHGEGKKGFFGWFNRMFEKSTHHYTDSVGGILRSTGR  540

MexB  541   YLLIYVVIVAGMIWMFTRIPTAFLPDEDQGVLFAQVQTPPGSSAERTQVVVDSMREYLLE  600
            YL++Y++IV GM ++F R+P++FLPDEDQGV    VQ P G++ ERTQ V++ +  Y L
AcrB  541   YLVLYLIIVVGMAYLFVRLPSSFLPDEDQGVFMTMVQLPAGATQERTQKVLNEVTNYYLT  600

MexB  601   KESSSVSSVFTVTGFNFAGRGQSSGMAFIMLKPWEERPGGENSVFELAKRAQMHFFSFKD  660
            KE ++V SVF V GF FAGRGQ++G+AF+ LK W +RPG EN V  +  RA   F   KD
AcrB  601   KEKNNVESVFAVNGFGFAGRGQNTGIAFVSLKDWADRPGEENKVEAITMRATRAFSQIKD  660

MexB  661   AMVFAFAPPSVLELGNATGFDLFLQDQAGVGHEVLLQARNKFLMLAAQNP-ALQRVRPNG  719
            AMVFAF  P+++ELG ATGFD  L DQAG+GHE L QARN+ L  AA++P  L  VRPNG
AcrB  661   AMVFAFNLPAIVELGTATGFDFELIDQAGLGHEKLTQARNQLLAEAAKHPDMLTSVRPNG  720

MexB  720   MSDEPQYKLEIDDEKASALGVSLADINSTVSIAWGSSYVNDFIDRGRVKRVYLQGRPDAR  779
            + D PQ+K++ID EKA ALGVS+ DIN+T+  AWG SYVNDFIDRGRVK+VY+      R
AcrB  721   LEDTPQFKIDIDQEKAQALGVSINDINTTLGAAWGGSYVNDFIDRGRVKKVYVMSEAKYR  780

MexB  780   MNPDDLSKWYVRNDKGEMVPFNAFATGKWEYGSPKLERYNGVPAMEILGEPAPGLSSGDA  839
            M PDD+  WYVR   G+MVPF+AF++ +WEYGSP+LERYNG+P+MEILG+ APG S+G+A
AcrB  781   MLPDDIGDWYVRAADGQMVPFSAFSSSRWEYGSPRLERYNGLPSMEILGQAAPGKSTGEA  840

MexB  840   MAAVEEIVKQLPKGVGYSWTGLSYEERLSGSQAPALYALSLLVVFLCLAALYESWSIPFS  899
            M  +E++  +LP GVGY WTG+SY+ERLSG+QAP+LYA+SL+VVFLCLAALYESWSIPFS
AcrB  841   MELMEQLASKLPTGVGYDWTGMSYQERLSGNQAPSLYAISLIVVFLCLAALYESWSIPFS  900

MexB  900   VMLVVPLGVIGALLATSMRGLSNDVFFQVGLLTTIGLSAKNAILIVEFAKEL-HEQGKGI  958
            VMLVVPLGVIGALLA + RGL+NDV+FQVGLLTTIGLSAKNAILIVEFAK+L  ++GKG+
AcrB  901   VMLVVPLGVIGALLAATFRGLTNDVYFQVGLLTTIGLSAKNAILIVEFAKDLMDKEGKGL  960

MexB  959   VEAAIEACRMRLRPIVMTSLAFILGVVPLAISTGAGSGSQHAIGTGVIGGMVTATVLAI  1017
            +EA ++A RMRLRPI+MTSLAFILGV+PL ISTGAGSG+Q+A+GTGV+GGMVTATVLAI
AcrB  961   IEATLDAVRMRLRPILMTSLAFILGVMPLVISTGAGSGAQNAVGTGVMGGMVTATVLAI  1019
```

Supplementary Figure 1. Alignment of *E. coli* AcrB and *P. aeruginosa* MexB.

Alignment of *Escherichia coli* AcrB (Ref. NP_414995.1) and *Pseudomonas aeruginosa* MexB (Ref. NP_249117.1) by BLAST. Identity 71%, positives 84%, Gaps 0%.

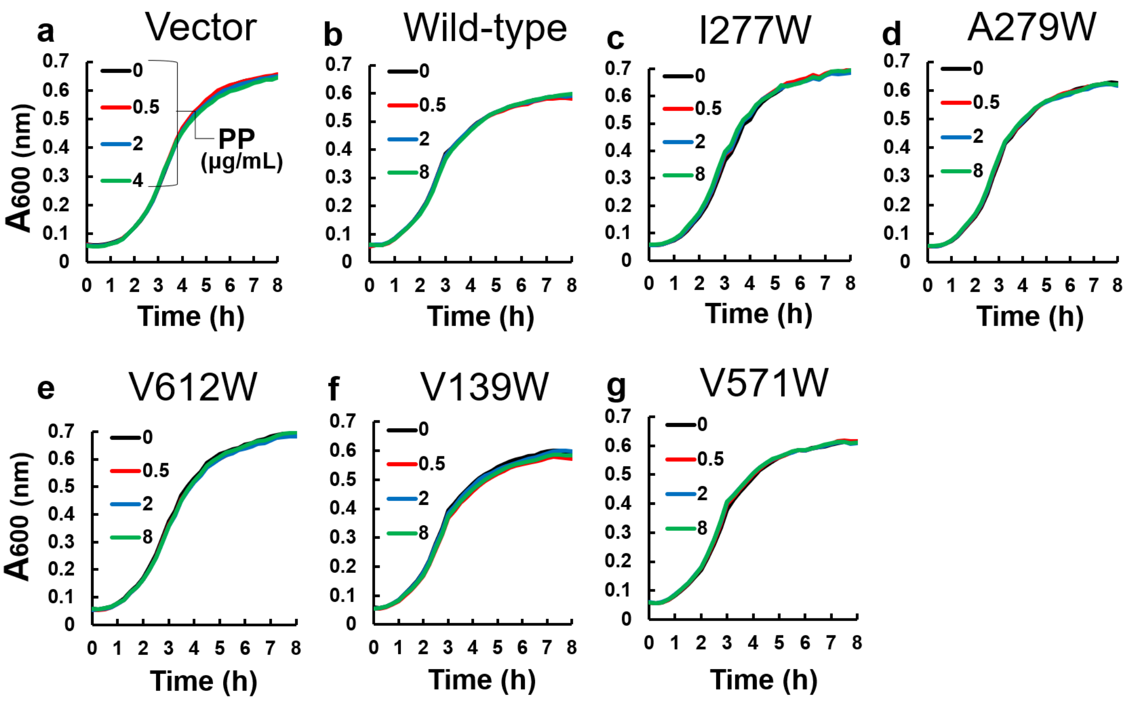

Supplementary Figure 2. Growth of *Escherichia coli* cells in the presence of various concentrations of ABI-PP without antibiotics

a The growth of *E. coli* MG1655Δ*acrB*Δ*tolC* cell harbouring the plasmid pMMB67HE in the presence of various concentrations of ABI-PP without antibiotics. **b-g** The growth of *E. coli* MG1655Δ*acrB*Δ*tolC* cells expressing wild-type MexB (b), MexB(I277W) (c), MexB(A279W) (d), MexB(V612W) (e), MexB(V139W) (f) and MexB(V571W) (g) in the presence of various concentrations of ABI-PP without antibiotics. These tests were performed in triplicates. Abbreviations: PP, ABI-PP inhibitor.

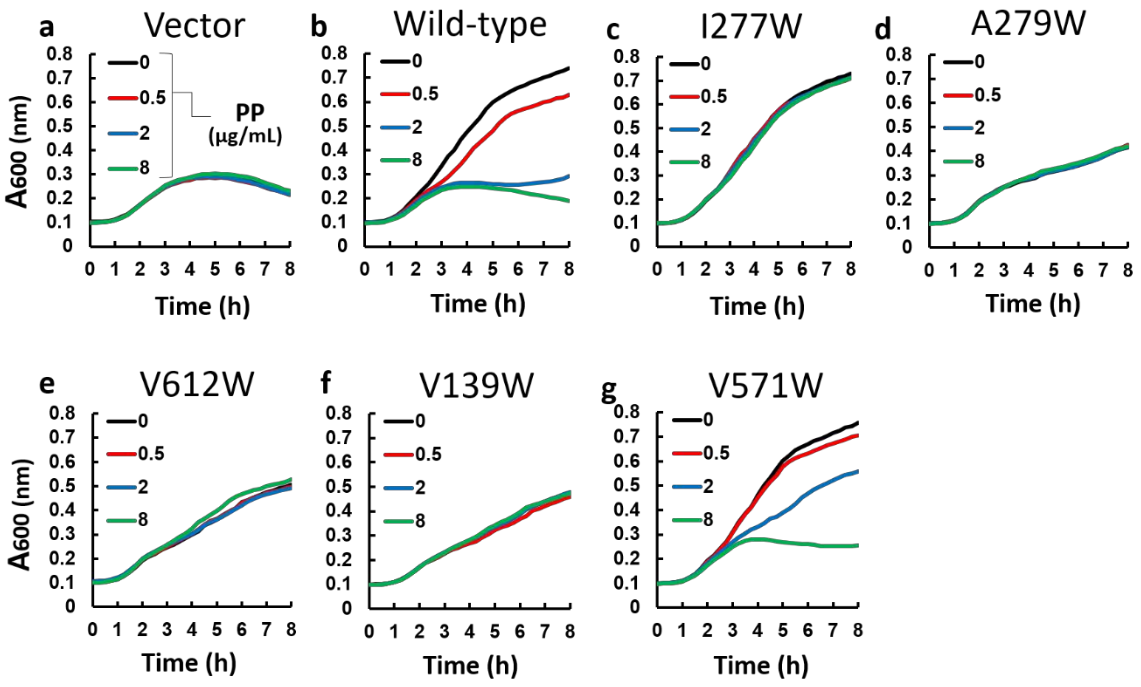

Supplementary Figure 3. Growth of *Escherichia coli* cells in the presence of 0.016 µg/mL of levofloxacin and various concentrations of ABI-PP

a The growth of *E. coli* MG1655Δ*acrB*Δ*tolC* cell harbouring the plasmid pMMB67HE in the presence of 0.016 µg/mL of levofloxacin and various concentrations of ABI-PP. **b-g** The growth of *E. coli* MG1655Δ*acrB*Δ*tolC* cells expressing wild-type MexB (b), MexB(I277W) (c), MexB(A279W) (d), MexB(V612W) (e), MexB(V139W) (f) and MexB(V571W) (g) in the presence of 0.016 µg/mL of levofloxacin and various concentrations of ABI-PP. These tests were performed in triplicates. Abbreviations: PP, ABI-PP inhibitor.

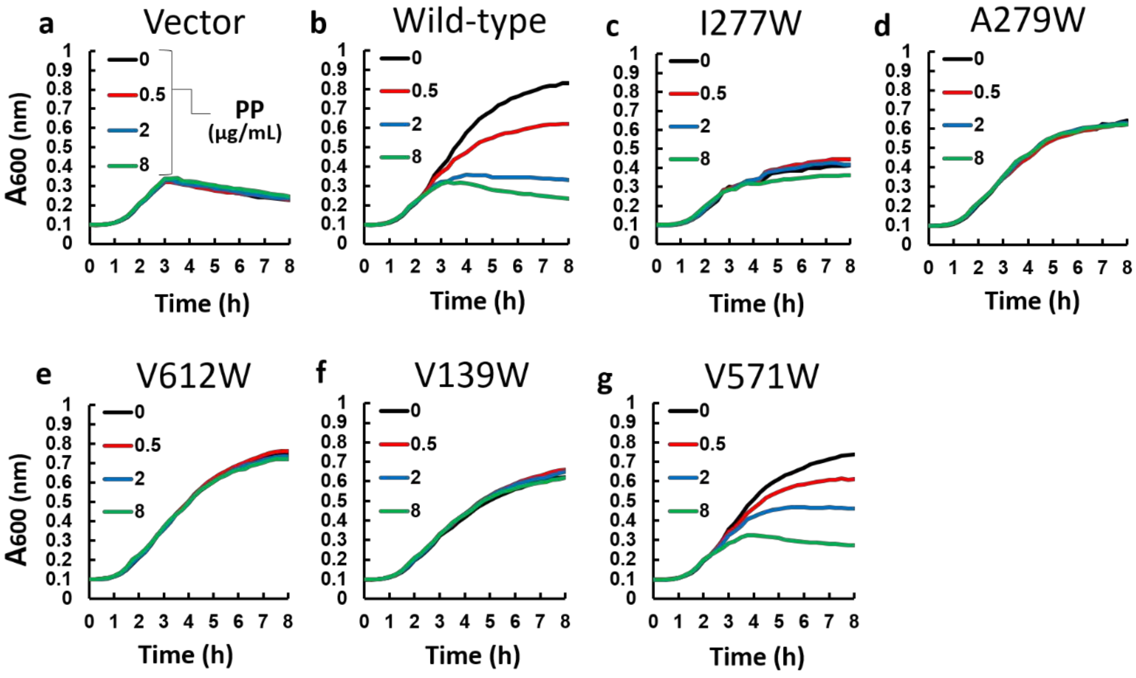

Supplementary Figure 4. Growth of *Escherichia coli* cells in the presence of 0.125 µg/mL of aztreonam and various concentrations of ABI-PP

a The growth of *E. coli* MG1655Δ*acrB*Δ*tolC* cell harbouring the plasmid pMMB67HE in the presence of 0.125 µg/mL of aztreonam and various concentrations of ABI-PP. **b-g** The growth of *E. coli* MG1655Δ*acrB*Δ*tolC* cells expressing wild-type MexB (b), MexB(I277W) (c), MexB(A279W) (d), MexB(V612W) (e), MexB(V139W) (f) and MexB(V571W) (g) in the presence of 0.125 µg/mL of aztreonam and various concentrations of ABI-PP. These tests were performed in triplicates. Abbreviations: PP, ABI-PP inhibitor.
